# Reflection at work – A conceptual model and the meaning of its components in the domain of VET teachers

**DOI:** 10.3389/fpsyg.2022.923888

**Published:** 2023-01-09

**Authors:** Mandy Hommel, Bärbel Fürstenau, Regina H. Mulder

**Affiliations:** ^1^Faculty of Electrical Engineering, Media and Computer Science, OTH Amberg-Weiden, Amberg, Germany; ^2^Faculty of Business and Economics, TU Dresden, Dresden, Germany; ^3^Faculty of Human Sciences, University of Regensburg, Regensburg, Germany

**Keywords:** reflection, informal learning, process of reflection, professional development, reflection at work, conceptual model

## Abstract

Professional development requires reflection. However, a conceptual model that considers the different perspectives on reflection remains missing. Regarding reflection, three different research streams can be distinguished: (I) an individual action-process-perspective, (II) a critical perspective, and (III) a social-relatedness perspective. From these three streams, important components are derived in the present study and integrated into one conceptual model. This model contains the individual and contextual components which influence reflection and considers reflection to be a process containing mutually influencing emotion, motivation, and cognition which can lead to various outcomes such as performance and, consequently, innovation. For illustrating the meaning of the model’s components in a specific professional context, we used data from an interview study with eight teachers of vocational schools. The conceptual model can serve as a basis for further research on reflection in all kinds of work contexts and be used to foster professional development, for instance by developing interventions to foster reflection.

## Introduction

Professionals in every field are faced with a multitude of challenges, such as globalization, digitalization, and changes in the conditions for work and work characteristics that emanate from such societal developments. Dealing with such challenges demands professionalism, which means knowledge, performance, and ongoing professional development (Messmann et al., 2010). This applies to all professionals, working in, for example, industry, agriculture, healthcare, the service sector, and education. Professional development is traditionally considered as involving participation in formal training. However, an important part of the professional development of people occurs during work (Felstead et al., 2009), which is often referred to as informal learning (Eraut, 2000). Informal learning can be defined as “any activity involving the pursuit of understanding, knowledge or skill which occurs without the presence of externally imposed curricular criteria” (Livingstone, 2001, p. 4). Informal learning occurs when it is required (Manuti et al., 2015), for example, in situations in which employees solve uncommon problems or face challenges and new tasks (Marsick and Watkins, 2001). In Eraut’s (2004) typology, reflection is a part of informal learning which is intended and is focused on past experiences, the so-called reactive learning (p. 250). Furthermore, Eraut (2004) distinguishes deliberate learning at work, which contains learning activities carried out on purpose and thus also reflection. Informal learning comprises both (work) action and cognitive activities, such as reflection, that accompany this action (Tannenbaum et al., 2010; Mulder, 2013). Regardless of the particular research stream, reflection is understood as establishing the link between action and outcome and creates the foundation for further learning and development (Tannenbaum et al., 2010). Thus, reflection is a specific form of thinking; a cognitive activity (Moon, 2006) closely connected with action in a specific context, in the sense of former and current action being the starting point and future action being the arrival point (Butler, 1996; Korthagen and Vasalos, 2005). As such, reflection is part of informal learning and contributes to professional development (Gustafsson and Fagerberg, 2004; Terhart, 2011; Naeve and Tramm, 2013; Richter et al., 2014; Messmann and Mulder, 2015; Kyndt et al., 2016; Sparr et al., 2017; Richter et al., 2020; Cattaneo and Motta, 2021).

Next to professional development, reflection can lead to other outcomes at different levels, for example at the individual level to self-development (Schön, 1983, 1987; Shulman, 1986), by gaining insights into the self and one’s own actions and thereby controlling, modifying, and further developing one’s action. At the team level, innovative work behavior (e.g., Messmann and Mulder, 2012, 2015; Widmann et al., 2016; Widmann and Mulder, 2018; Kmieciak, 2020) is a possible outcome, and at organizational level, changes in management policy, and organizational development are outcomes (Pässilä and Vince, 2015). Numerous studies, theoretical and empirical, regarding the concept of “reflection” and notions of reflection already exist (Skovsmose, 2006). The perspectives and foci of these studies differ according to the streams and traditions upon which research on reflection is conducted. Nevertheless, the different perspectives on the streams can be drawn upon to better understand the process of reflection and its complexity. Based on these perspectives, a conceptual model of reflection can be developed that integrates the different perspectives and combines their components. Reflection requires acknowledgement as a process that is caused by triggers and influenced by other factors, and that has certain outcomes. Such a conceptual model of reflection can be used as a profound basis for the analysis of the value of existing research and can provide entry points for further research.

Therefore, our *aim* is to develop a conceptual model of reflection by deriving components from the different perspectives and integrating them into one model taken into account a few considerations. First, that reflection is a process running from triggers that are used for the intrapersonal process which leads to outcomes such as performance and consequences for further action and future reflection. Second, the notion that the intrapersonal process as such is complex. Third, such a model must also contain other influencing components and all relationships among them. In addition to the development of a conceptual model, we aim to illustrate the meaning of the model’s components exemplarily. For this purpose, we have chosen the professional field of teachers in vocational education and training (VET) because of the characteristics of their work and conducted in-depth interviews with VET teachers regarding their subjective views on reflection in and on everyday work situations. Teachers, in general, need to be specialists in teaching and learning, take on educational tasks as well as assessment and advisory tasks, participate in school development, and constantly develop their competences (KMK Kultusministerkonferenz, 2019). In the VET context, the situation is also challenging as teachers face culturally diverse classes of students, whom they must prepare for the constantly changing workplace conditions. Teachers must act as role models for their students as future professionals and therefore have to be up to date regarding workplace developments. For teachers as for other professionals, reflection is a prerequisite for professional development and for successful teaching (Kuhn et al., 2018, p. 339; Shulman, 1986; Körkkö et al., 2016). Against this background, the following research questions guided our work:

Research question 1: What are the pivotal components in reflection processes?

Research question 2: What is the meaning of the model’s components for a specific domain, in this case the professional field of VET teachers?

First, we elaborate on the essential approaches of reflection and thus lay the foundations for the development of the conceptual model (2). We subsequently introduce the empirical part of this study, sample, data collection, analysis (3), and results (4) we used to illustrate the model’s components. We conclude by reviewing limitations and prospects.

## Development of a conceptual model

Below, we first introduce the different streams regarding reflection, each of which can be associated with a specific perspective that will be elaborated on. We then derive from these perspectives the components that are incorporated into a conceptual model and explain them in detail.

### Different approaches towards reflection

The work of Dewey can be considered the common origin of streams in research on reflection (Rodgers, 2002; Høyrup and Elkjaer, 2006; Kyndt et al., 2018). For Dewey, reflection can be understood as “active, persistent, and careful consideration of any belief or supposed form of knowledge in the light of the grounds that support it, and the further conclusions to which it tends” (Dewey, 1910, p. 9). Reflection can help to get insight into actions as well as control, and modify actions, and lastly develop new actions. The research streams on reflection (e.g., in philosophy, educational science, psychology) developed since Dewey can be distinguished between an individual action-process perspective (I), a critical perspective (II) and a social-relatedness perspective (III) (Høyrup and Elkjaer, 2006).

The individual action-process perspective (I) is based on Schön (1983). Although Schön’s ideas are sometimes considered a second tradition alongside (Anderson, 2020), the roots of Schön’s practice−/practitioner-oriented approach can be traced back to Dewey. Focusing on the professional action of a practitioner, Schön (1983) distinguishes reflection during and after an action (reflection in-action and reflection on-action). In addition, with ‘reflection before action’ a third time perspective can be elicited that is already apparent in Dewey’s (2001) work, whereby reflection is “the relation between what we try to do and what happens in consequence” (Dewey, 2001, p. 150), which is also immanent in the cycle of action. The ALACT model (Korthagen and Vasalos, 2005, p. 49) represents the cycle of action to structure the reflection process by means of a tool using guiding questions to support professional development in teacher education. The model is particularly suitable for analyzing one’s own past actions as a teacher in a specific situation, becoming aware of certain aspects, which can be used to develop and implement alternative actions. Although in the development phase we focused on teacher action, specifically the process idea of the model should be part of a conceptual model suitable for all kinds of professions. In studies categorized by this individual action-process perspective (I), reflection is considered a process initiated by different triggers, influenced by other factors in its course, and leading to certain outcome(s) (Korthagen and Kessels, 1999; Høyrup, 2004; Korthagen and Vasalos, 2005; Hommel and Clarke, 2015). Due to the emphasis on mainly cognitive aspects (Zembylas, 2014), research so far did not adequately consider all three psychological components (emotion, motivation and cognition) of reflection processes, nor their interactions.

The critical perspective (II) emphasizes the questioning of premises for problem-solving as the content of reflection (Mezirow, 1998). In addition to the premises, the object of reflection can be the content or processes of problem-solving. This perspective highlights how reflection is always content related, and it therefore matters what the characteristics of the content are. This broadens the view from the individual to the social perspective (Reynolds, 1999; Van Woerkom, 2004; Høyrup and Elkjaer, 2006, p. 35). However, it specifically focuses on critically analyzing and justifying the premises upon which problems are based (Mezirow, 1990). In the context of levels of reflection (e.g., Hatton and Smith, 1995; Moon, 2004; Korthagen and Vasalos, 2005; Carroll, 2010; Schley and Van Woerkom, 2014), which are differentiated according to various criteria, critical reflection is usually considered qualitatively as a sophisticated level of reflection (Mezirow, 1998; Moon, 2004). A key benefit of this line of thinking is that it matters what is reflected upon (e.g., Messmann and Mulder, 2015). In the context of job characteristics, for example, Kmieciak (2020) investigated autonomy, intensity and problem solving regarding critical reflection. Thereby, critical reflection was distinguished from reflection as a “more profound, advanced and demanding form of reflection” (Kmieciak, 2020, p. 440). A positive relationship between problem-solving, concerning jobs that require dealing with unknown problems, finding creative and unique solutions, and critical reflection was shown (Kmieciak, 2020). For the other two characteristics (autonomy and intensity) no relationship was found. The results underline that socio-physical and spatio-temporal context components affect reflection and should be considered when modelling reflection as a process.

Thirdly, the social-relatedness perspective (III) puts reflection into social practice and emphasizes reflection as a collective process. From this perspective, reflection is considered an individual phenomenon, but the complexity is expanded by considering the social context and possible joint reflective activities. Situational influences must therefore be considered, such as one’s own behavior, the perceived behavior of other people, or other occurrences that can be used as triggers for reflection (Dalsgaard, 2020). Reflection processes, such as individual activities, are embedded in a social context. Although reflection is an individual activity, it can be performed together with others, concerning collective reflection (Raelin, 2002; Schley and Van Woerkom, 2014; Foong et al., 2018; Prilla et al., 2020). Collective reflection can be considered a social learning activity that occurs together with others and thus influences the intrapersonal process of reflection. Individual reflection is not a social learning activity, but the individual process of reflection can occur in a social context and be influenced by the characteristics of the context.

Taking the three perspectives into account, we define reflection as a conscious and systematic process of elaborating meaning and deepening understanding of a specific content that interrelates cognitive, emotional, and motivational elements; is based on work actions, experiences, and knowledge; takes place in a social context and contributes to individuals’ professional development (Hommel and Clarke, 2015, p. 1,718).

### A conceptual model on the process of reflection

**Figure 1:**
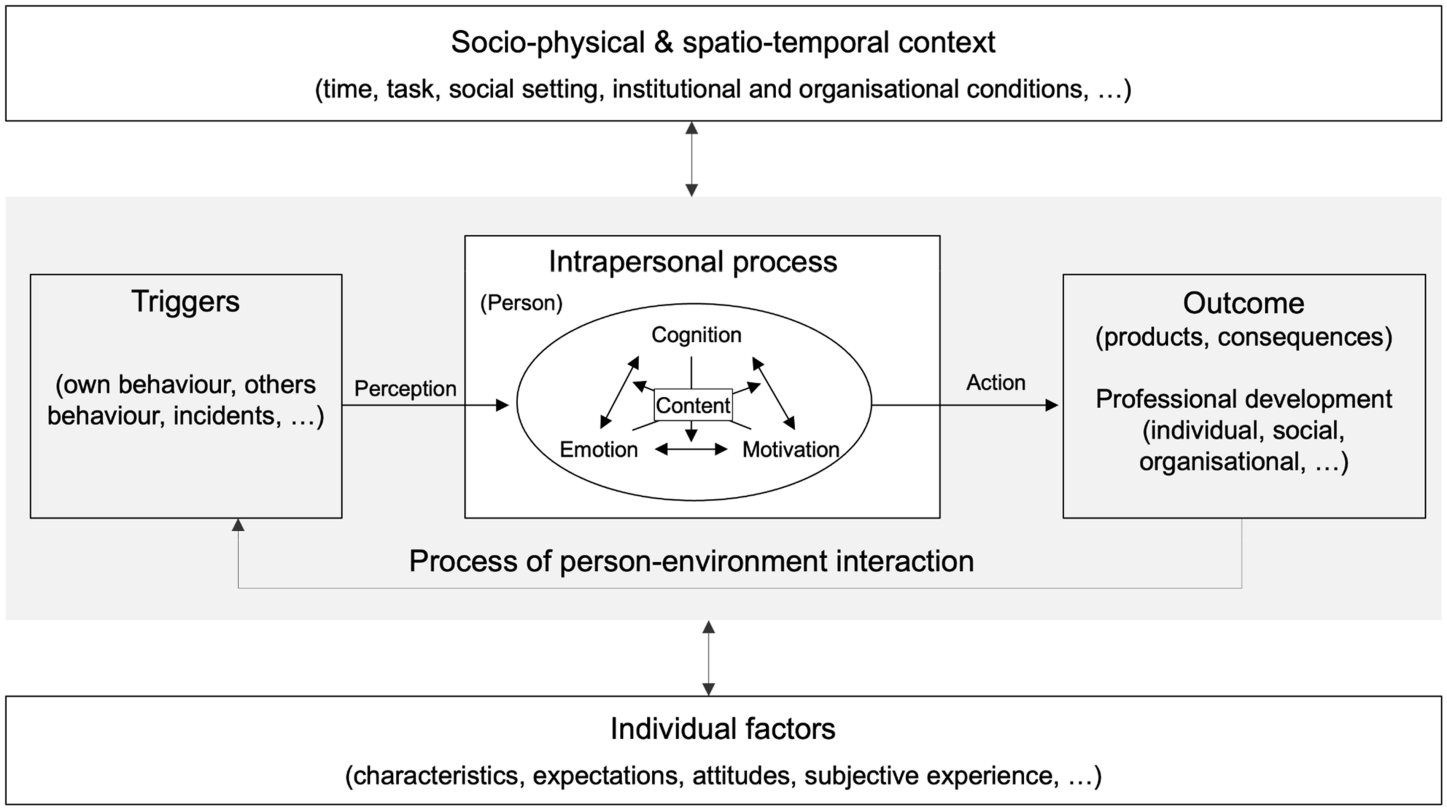
Conceptual model of reflection (adapted from Hommel et al., 2020, p. 2; based on Becker et al., 1987).

By modelling dynamic and complex reflection processes, hints for overcoming obstacles and clues for supporting reflection behavior can be identified. The course of reflection can be visualized in a simplified manner by using an I-P-O (input, process, output) model (Biggs, 1993) that helps to conceptualize, describe, and analyze reflection as a process with its triggers, intrapersonal processes and outcomes, and its different influencing factors, which shows its complexity. In addition, complexity is in the intrapersonal process, which consists of different mutually influencing components (motivation, cognition, and emotion). Furthermore, this model should be understood as a dynamic one in the sense that all components can influence each other at all times (Figure 1).

From the different streams of research on reflection we derived relevant factors related to the individual and their actions that become the object of reflection. Beginning with the **individual action-process perspective (I)**, the components of the *intrapersonal process of reflection* can be identified. As an intrapersonal process (Høyrup and Elkjaer, 2006), reflection is considered a cognitive activity (Hetzner et al., 2011). Research in this perspective (I) acknowledges that emotion plays a role in reflection (Boud et al., 1985). However, it is mainly the attending to emotions that is meaningful regarding the triggers for and the aims of reflection in this perspective. Negative emotions should be overcome, and positive emotions should be used for the further development of the individual (Høyrup and Elkjaer, 2006). Thus, cognition and emotion as psychological processes are considered. Lacking so far is motivation, as a third component, and the interactions between these three components.

We consider reflection an individual process of person-environment interaction (Becker et al., 1987). The person perceives details of the environment which s/he processes internally. The process of perception and further processing in the form of reflection can be initiated by several *triggers*. Uncertainty, for example, can trigger reflection (Dewey, 1910). Cognitive conflicts or, per Piaget (1977, p. 91), “disturbances,” are also triggers for reflection. In both cases, people feel confronted with the feeling of uncertainty in a situation caused, for example, by something unknown; new problems or strategies that no longer seem to work. These triggers are always situated and are perceived and processed individually. Although reflection is genuinely considered a cognitive activity (Dewey, 1910; Hetzner et al., 2011), we argue that during the reflection process cognition, emotion, and motivation influence each other. Here we integrate the subsystem of psychological components (cognition, emotion, and motivation) from the model of Becker et al. (1987). Since humans can be regarded as system of systems, it is necessary but not sufficient to consider the psychological system composed of components, such as cognition, emotion, and motivation. Aiming at developing a holistic model of factors influencing reflection, we need to also consider the person’s physical system. The physical and psychological systems mutually interact (Becker et al., 1987). Conditions, such as being tired, ill, or in pain, can be assumed to influence reflection.

The reflection process is influenced by *socio-physical and spatio-temporal conditions*, and individual factors. Spatio-temporal conditions may be time constraints or work tasks that require solving. Socio-physical conditions are, for example, the social setting and interaction, as can be derived from the **social-relatedness perspective on reflection (III)**. There is evidence that these relationships and mutual influences are complex. They can for instance affect each other positively or negatively, the direction of the influences can change, and the intensity of the influences can change over time (e.g., Watzek et al., 2022). For example, Watzek et al. (2022) found effects of positive emotions on team reflection, and positive effects of team reflection on positive emotions. Triggers for reflection can be found, for example, in errors and feedback which cause reflection (Van Woerkom and Croon, 2008). Others are related to “shared sense-making and collaborative engagement” (McArdle and Coutts, 2010, p. 201) that reveal problems of comprehension or misconceptions. The socio-physical factors influencing the reflection process include job characteristics such as job demands, autonomy, job variety, and constraints but also other workplace-related characteristics such as leadership (Anselmann and Mulder, 2020), access to resources or opportunities for collaboration (Kyndt et al., 2016), learning culture (Barnett and O’Mahony, 2006), feedback culture (London and Smither, 1995; Mulder and Ellinger, 2013), error culture (Bauer and Mulder, 2007), or team climate (Hetzner et al., 2011). It is assumed that aspects of the feeling of safety in the environment, for example in relation to errors and feedback in addition to psychological safety are important for supporting reflection processes (e.g., Anselmann and Mulder, 2018). All these aspects are considered contextual aspects that can promote or hinder reflection.

*Individual factors* influencing the reflection process are relatively stable components such as attitude which tend to be traits. Furthermore, a wide variety of individual characteristics influence reflection (Kyndt et al., 2016), such as the knowledge, skills, competences, and experiences of a person. Other important aspects are attitudes such as self-efficacy, commitment, and values or interest (Yost, 2006). Expectations, subjective views on reflection, as well as background characteristics such as age, gender, and career stage, can also affect reflection. Initiated by triggers, influenced by individual factors and spatio-temporal as well as socio-physical conditions, the process of reflection is considered to depend on a person’s physical state and the interaction between cognition, emotion, and motivation in the process.

The important component to derive from the **critical perspective (II)** is the notion of the *content* of reflection. Reflected is upon specific content, i.e., ‘what does someone reflect on.’ While reflecting, one is engaged in activities, such as analyzing, valuing (Kyndt et al., 2016), elaborating, and critically evaluating the content, the processes, or the premises of problem-solving (Mezirow, 1990). The content of reflection can be the task, the social context (e.g., co-operation with colleagues), or one’s own performance (Messmann and Mulder, 2015). In the sense of critical reflection, the premises of problem-solving in its respective (historical, social, cultural, and political) context can be reflected on (critical reflection, e.g., Mezirow, 1990, 1998; Van Woerkom, 2003).

Regarding the *outcomes of reflection*, there is empirical evidence that reflection can lead to, for instance, knowledge or skills (Van Woerkom, 2003; Boud et al., 2006; Ellström, 2006), but also initiate further (intended) action (Aebli, 1980), as for instance engagement in learning activities that can lead to further professional development (Figure 1). In addition to the effects of reflection at the individual level, it can also affect performance or the innovative work behavior of work teams (e.g., Widmann et al., 2016). Such examples of empirical evidence on specific connections between certain components remain exemplary and do not cover the whole model.

The complete process can run as a cycle, more likely even as an iterative or chaotic process (Marinova and Phillimore, 2003), as the process can integrate results of previous reflection processes and is influenced by new occurrences that can be used as triggers. Furthermore, the complexity and dynamics are caused by changing situations and their specific characteristics, such as changes in content or components of the context.

Analyzing professionals’ reflection processes increases insight into real reflection processes, how reflection is caused, and the outcomes that can be achieved. Therefore, we developed a conceptual model that can be used in empirical studies for analyzing reflection processes. The value of the model is that components derived from the various streams are integrated, that reflection is considered a process in multiple ways (reflection process with triggers, intrapersonal process, and outcome; and within the intrapersonal process emotion, motivation, and cognition interacting) and that it is considered a dynamic and complex process. The model can be used as a framework for analyzing the process of reflection in specific domains by determining the meaning of the model’s components.

## Interview study

To illustrate the meaning of the model’s components, a qualitative interview study was conducted by asking VET teachers for their subjective views on reflection in their professional work context. Those subjective views are important as they have different functions, such as defining situations and reconstructing reality, explaining and predicting events, and generating action plans and action recommendations (Dann, 1983). Subjective views have an action-accompanying, action-controlling and action-guiding character (Laucken, 1974); they influence action and are influenced by action (Schwarzer and Schwarzer, 1982). Subjective views are not readily visible from the outside, but must be explicated by the person. S/he has to be supported in this explication process. Open or semi-structured interviews are one method of achieving this.

### Sample

The participants were eight teachers (7 f, 1 m), all working at the same vocational school for business and technology in Germany. They were on average 50 years old (*SD* = 8.2) and had on average 20 years of teaching experience (*SD* = 7). Each teacher taught one up to three different subjects, among them business, engineering, English language, German language, sports, and mathematics. The eight teachers can be regarded a convenience sample. Since we do not aim at generalizability of the results, which would be linked to the criterion of representativeness, the material used here does not have the status of empirical data, but that of an illustration (Helfferich, 2011, p. 172).

### Data collection

The teachers received an email prior to the interviews explaining the topic and the objective of the study, namely, to identify their subjective views on their own reflection in the context of their professional work behavior, specifically related to teaching. Semi-structured in-depth interviews were conducted on two consecutive days on site in an empty classroom, i.e., in an environment that was authentic and familiar to them. The interviews were conducted anonymously, i.e., interviewer and interviewee did not know each other by name. For the salutation and the conversation, the participants used the common German forms of politeness, however no fake or code names. This approach enabled the interviewees to answer freely and without bias. The participants reported great feelings of security due to the opportunity to remain anonymous. On average the interviews lasted 52.1 min (*SD* = 8). One interviewer conducted the interviews. The interviewer was trained beforehand by carrying out two test interviews. The test interviews were conducted with two interviewers using test persons comparable with those of the convenience sample. On interviewer was responsible for guiding the interview, the other one for observing and recording the process. The test interviews served to detect and avoid errors (e.g., with regard to the setting up of the situation and the questions).

The interview guide refers to the components of the conceptual model (Figure 1). At the beginning, the goal of the interview was clarified, namely that the research group is interested in teachers’ views on reflection associated with their everyday teaching. We used everyday language to explain our goal. In accordance with our theoretical background, we explained reflection as a conscious consideration of all factors relevant in the professional work context, specifically teaching. No further definition was given as not to limit our subjects’ exploration of the topic. The first question accorded with the Critical Incident Technique (Flanagan, 1954), asking the respondent to describe the concrete situations that led to reflection. Teachers were asked to recall and to describe situations in which they had reflected on their teaching (reflective situation). This general description of the reflective situations served as a non-controlled warming-up and explication aid as well as to identify all components of the model and beyond that teachers reflect upon without pushing them in a direction determined by the interviewees. In this manner, subject-object confounding could be avoided.

Following the open beginning, guiding questions were asked about the main components of the model (Figure 1). In the following we explain the intention associated with the guiding questions instead of just listing the questions:

1. *Triggers*. We asked teachers to name triggers in order to find out which factors they perceived that set the reflection process into motion. Both personal and situational factors might serve as triggers.
2. *Cognition* as an intrapersonal process component covers teachers’ subjective explanations concerning what causes situations reflected upon and what results from those situations. Causes as part of the explanations are closely related to triggers. The difference is that triggers refer to perception whereas cognition refers to the explanations of what is perceived.
3. *Emotion* as an intrapersonal process component represents the state of a person’s psychophysical system, such as a bad or good mood, or a concrete emotion such as fear or anger that a person uses during reflection. To get insight into this state, we asked for the feelings associated with reflection.
4. *Motivation* as an intrapersonal process component addresses the aims and expectations associated with reflection. Therefore, we attempted to identify the role of motivation in the course of reflection.
5. *Outcome* is regarded as action and the results of actions taken. Action refers to measures taken to enhance reflection, or reach the aims associated with reflection. In addition, we asked teachers to consider the potential consequences of their actions.
6. *Socio-physical and spatio-temporal context* might be seen, for example, in time and location of reflection, or in factors influencing reflection, such as a reflection-friendly school climate. Teachers were asked to describe the conditions of reflective situations and to name factors which promote or hinder their reflection.
7. *Individual factors* such as attitude toward reflection or personal characteristics can influence reflection. Therefore, we asked about what personal factors teachers regard as factors influencing reflection.

### Data analysis

All interviews were audio-recorded. The audio files were transcribed to facilitate data analysis. Qualitative content analysis was applied, which is a systematic, replicable technique for assigning meaningful statements to content categories based on explicit rules of coding (Krippendorff, 2018). In the course of a content analysis aiming at structuring material, categories can be derived from theory, or literature which provide a grid for assigning empirical material to pre-defined categories. In contrast, if no grid is available, categories must be derived inductively from the data in the course of summarizing original statements (Mayring, 2015). Here a mixture of structuring and summarizing was applied. We began with main categories derived from the interview guide, followed by inductively gaining coding units from the transcripts (Kuckartz, 2018). We summarized the coding units to subcategories which we assigned to main categories. This resulted in a category system valid for our set of data. The category system forms the central instrument of the qualitative content analysis, so that category construction and justification are the focus (Mayring, 2015). The assignment of text passages to categories is systematically carried out by rule-guided interpretation. In the analysis phase, the rules may be revised and adjusted in feedback loops, but for a final material pass they remain constant. To ensure consistent coding, we developed a coding guideline which provides definitions, anchor examples, and coding rules for all categories (Table 1). Then, a second coder used the guideline to recode the material. The differences were discussed and agreed upon between the two coders to develop a common understanding.

**Table 1:** Extract from coding guideline.

Category	Subcategory	Definition	Anchor example	Coding rule
Triggers	Triggers of reflection (social interaction)	Exchange with persons that takes place or questions that are asked.	when my boss [...] is interested in my teaching and asks about it (RBS01 lines 464–466)	Social exchange with others that triggers reflection.
Emotion	Disappointment	Person is disappointed in the reflected situation	And I am often very disappointed about the students. (DBS01, lines 231–232)	The emotion of disappointment is expressed.
Outcome	Lessons – linguistic change	Lesson contents are prepared (linguistically) differently	But that students...understand my language, I accommodate the students and like to formulate it sometimes colloquially (DBS08, lines 312–313)	A change in verbal communication is expressed.
…	…	…	…	…

Using the final coding guideline, we recoded the individual transcripts and subsequently assigned coding units to the respective category. Two researchers coded the material independently. The differences were discussed and agreed upon between the two coders. Based on the analysis qualitative conclusions regarding teachers’ views on reflection and our model (Figure 1) are possible.

## Results

The results of the analysis indicate that teachers have a differentiated view on reflection. Consequently, our coding system comprises a variety of main and subcategories, filled with diverse coding units, respectively, statements. An overview of main and subcategories and corresponding explanations is presented below. In addition, the categories are illustrated by original statements (Table 2).

**Table 2:** Original different things. Also parents. Increasingly, I have views on reflection.

Category	Subcategories	Statements
(1) Triggers	Perceived positive or negative deviations (planned – actual instruction)	Either something worked out wonderfully, or the expectations you had, the goals you had, were not met (T2I1, lines 390–393)
	Perceived emotion	When I am dissatisfied with my own teaching (DBS01, line 19–20)
	Perceived student behavior and achievement	What I realized in a very negative way is that the problematic situations stay in your memory. In other words, the students who made things difficult for you or where you are not completely satisfied with yourself (T1I4, lines 13–15)
	External prompts	Triggers for me are also...different things. Also parents. Increasingly, I have parent inquiries (DBS08, lines 373–374)
(2) Cognition	Causes and effects of deviations between planned and actual instruction	When the students do not show the kind of activities or reactions that I have calculated for or that I expected. (DBS01, lines 225–227)
	Causes and effects of teacher action and achievement	So there are sometimes situations where you say: “You’ve mastered that”. [...] That gives you strength to do new things again, because you have mastered situations that others did not have to master. (T2I1, lines 295–300)
	Causes and effects of reflection	And then there are sleep disorders or something. (DBS01, line 16)
(3) Emotions	Positive emotions	I do not want to say pride. Yes, pride in the student (DBS08, line 205–206)
	Negative emotions	Then I’m annoyed when I say “Gee, BS3, that was a load of rubbish” (RBS02, lines 96–97)
(4) Motivation	Improvement of oneself	As a teacher, I think you have to constantly work on yourself. I think that is very important (DBS01, lines 578–579)
	Improvement of instruction	In any case, the goal is that I try to find out...at least an explanation for the situation and possibly also a possibility of change (DBS04, lines 520–521)
(5) Outcome	Measures to improve instruction	I then included this in the lesson, because I also thought the question was important (DBS02, lines 75–76)
	Measures to improve reflection	That I also make a note to myself, “Pay attention to this.” (DBS01, lines 637–638)
	Consequences of measures to improve instruction	There is feedback from the students that individual contents have helped them a lot (RBS01, line 338–339)
	Consequences of measures to improve reflection	And then also actually...also others have reflected (DBS02, lines 76–77)
(6) Socio-physical and spatio-temporal context	Time resources and workload	And what prevents me from reflecting is above all this burden. The many hours that you have and that you have the feeling that you have to rush through the material over the day, over the week, and that there is very little time to internalize and think through the whole thing (DBS01, line 757–760)
	Work climate	If my boss or just anyone is interested in my teaching, asks about it (RBS01, lines 464–465)
(7) Individual factors	Reflection habits	So basically I look at the lessons again afterwards. Logically (RBS02, lines 122–123)
	Importance of reflection	So clearly, a teacher must always self-reflect. That is important (RBS02, line 66–67)

The respondents mentioned manifold situations of reflection. They essentially concern instructional situations, for instance a lesson’s introduction or content, the quality of its preparation, time allocation for content, or methods used. In addition, teachers consider their planning schedule in the sense of what to plan when. Closely related to instruction, teachers question/reflect upon themselves as persons, especially their perception by the students. Another incident is the request to reflect, such as the case in the interview situation or in communication with others.

Furthermore, the report of critical incidents comprised situational features of reflection situations, such as the frequency of reflection (e.g., teachers report that they reflect often), time of reflection (e.g., before, after, or during a lesson, in/on actions during a lesson, in the evening, directly before or after work, in a break between two lessons, in leisure time, at the beginning of the school year, before or after exams); social setting of reflection (e.g., either alone, together with peers, together with students, together with a partner outside school, or together with a supervisor); or location of reflection (e.g., at home, in school).

### Triggers

As triggers for reflection teachers name the interview situation. However, teachers mainly mention instruction-related triggers for reflection:

- *Perceived positive and negative deviations* as well as consistency between planned and actual lesson.
- *Perceived emotion*, both positive (joy about or satisfaction with a lesson) or negative (discomfort or disappointment in a lesson).
- *Perceived student behavior and achievement* (e.g., knowledge and performance level, especially when it is not sufficient, or classroom disputes).
- *External prompts* (e.g., social interactions with colleagues or questions colleagues raise upon instruction or related topics, the need to prepare a lesson, reading literature, daily events, or questions from parents).

### Cognition

Regarding reflection as a cognitive process, teachers mentioned especially thinking about cause-effect relationships related both to instruction and reflection. However, it was not always the case that both causes and effects were mentioned. Some examples for cause-effect relationships are:

- *Causes and effects of deviations* between planned and actual instruction (e.g., incorrect time estimation leads to not getting through the content).
- *Causes and effects of teacher action and teacher achievement*, for example, teaching that is successful due to good preparation, which in turn results in contentment (For this example, cognition and emotion are interlinked).
- *Causes and effects of reflection*, whereby causes mainly equal triggers (see above), and effects are for instance (a) learning from errors such as uncovering weaknesses in one’s own actions, (b) balancing and self-positioning (e.g., teachers conclude that things are going well and they should doubt themselves less), (c) planning future teaching (e.g., putting fewer demands on the students), (d) physical problems (e.g., sleep disorders as consequences of reflection) (These examples show that cognition and action (outcome) are closely related).

### Emotion

Emotions in the course of the reflection process are usually equated by teachers with emotions related to instruction. Teachers re-feel the emotions originally associated with teaching again in the reflection process. They report about positive and negative emotions whereby negative emotions seemed to be more prominent:

- *Positive emotions* such as to joy, contentment, or pride due to successful instruction.
- *Negative emotions* such as dissatisfaction with a lesson, uncertainty about correct classroom behavior or the feeling of being questioned by a student (New situations such as teaching new classes can lead to uncertainty. Students’ unproductive social behavior which cannot be managed by the teacher can cause frustration, and a bad classroom climate can lead to discomfort).

### Motivation

Aims and expectations related to reflection represent the motivational component in the reflection process. Among the aims identified are the following:

- *Improvement of oneself*, for example becoming a better teacher.
- *Improvement of instruction* in a manner that supports students optimally.

### Outcome

Outcome comprises action/measures teachers taken to either change instruction or change reflection as well as the (expected) consequences of these measures.

- *Measures to improve instruction,* for instance the change of the amount of content to be taught, or the change of instructional methods (e.g., not teaching exclusively from the front of the classroom, writing down more notes on the blackboard, including more lesson breaks to give students the chance for some physical exercise).
- *Measures to improve reflection,* for example utilizing feedback from third persons (students, colleagues, or supervisors) to stimulate own reflection, engaging in self-evaluation by jotting down thoughts in bullet form, talking with colleagues and deliberately taking more time for reflection.
- *Consequences of measures to improve instruction*, for instance better motivation of students, better performance of students such as better transfer of knowledge to new situations and tasks.
- *Consequence of measures to improve reflection*, for example others were also stimulated to reflect.

### Socio-physical and spatio-temporal conditions

Teachers mentioned certain factors influencing reflection in a positive or negative manner:

- *Time resources and workload* which means that teachers would prefer to have more time for reflection and spend less time on additional tasks (e.g., school conferences, parents’ evenings).
- *Work climate* which means that an intact relationship among teachers is regarded as beneficial for reflection.

### Individual factors

Teachers mentioned only a few individual factors influencing reflection. One factor is reflection habit; the other the subjectively assessed importance of reflection.

- *Reflection habits*, which refers to that teachers think their habits influence reflection and that, in general, people might differ in that they reflect regularly or on occasion.
- *Importance of reflection* which means teachers in general regard reflection as very important.

## Conclusion

Our contribution resulted in a conceptual model on reflection that can be used to analyze reflection processes at work to increase understanding in these processes. In doing so, we derived and integrated relevant components of different streams. In addition to this exclusive feature, the model captures considering reflection as a process. First, it runs from triggers, through to the intrapersonal process and leading to outcomes, but this is not considered a linear process *per se*. The relationships between the components can run, at all times, in all directions. Secondly, the process aspect is captured in the intrapersonal process itself, where not only is cognition seen as part of reflection, but also emotion and motivation and, moreover their mutual, non-linear relationships. Moreover, in the figure the other influencing factors are included in ‘socio-physical & spatio-temporal context’ and ‘individual factors.’ Beyond existing conceptual models (Korthagen and Kessels, 1999; McAlpine et al., 1999; Balla et al., 2009; Dalsgaard, 2020), these considerations lead to a new conceptual model, which captures the dynamics and the complexity of reflection processes (Mulder, 2022).

This model can be used for reflection processes at the individual level of one person and also can be used for even more complex work settings such as teams (e.g., colleagues working together, Balla et al., 2009; Fluijt et al., 2016). In an interview study, we focussed at the individual level of VET teachers, whereby we discovered the meaning of the components in the model in actual work situations. The data indicate that VET teachers’ subjective views on reflection can be very diverse. They expressed a variety of aspects associated with reflection, such as triggers, outcomes or for instance emotions in reflection processes. Furthermore, teachers had difficulties to clearly distinguishing reflection about their teaching and reflection about their reflection processes.

This outcome underlines the complexity of reflection processes and the consequential challenge of analyzing these. We experienced that the use of our conceptual model as a framework for analyzing reflection processes in concrete work contexts was appropriate and effective because it contains all relevant components and captures the actual complexity in real working life.

### Limitations and further research

The outcome of the interview study might be due to the specific context, as well as the competences of the interviewers. Answering interview questions (which requires reflection) regarding reflection (content to be reflected on) is a meta-cognitive challenge for interviewees as it requires reflection upon reflection, and not reflection upon instruction. Interviewers should be trained in this regard to avoid unintended (con)fusion.

The interview study was an initial attempt to determine the usability of the conceptual model and illustrate the meaning of the model’s components in a first professional field. In doing so we were able to gain insights into the meaning of the components of the reflection processes at work. Although insight in reflection in VET teachers’ work—as one specific domain—was gained, we suggest using larger samples in further studies. Furthermore, this study focussed on a specific part of the work of VET teachers’ work, namely teaching. Therefore, further studies are required to study reflection in other important parts of these teacher’s work (KMK Kultusministerkonferenz, 2019).

Although, we carried out this interview study in one specific domain, the conceptual model has been developed to be used in all kinds of professional domains. Therefore, further studies on reflection processes can be conducted in different domains, and with different jobs with different tasks, to increase insight in the meaning of the components. In addition, it can be used to compare jobs and domains in the future. Further empirical evidence from different work contexts can lead to strengthen the value of the model.

Next, to the focus on one domain, so far this interview study has revealed a specific part of the conceptual model, namely the meaning of the different components. Studies that capture more the complexity and go beyond analyzing the meaning of the separate components by investigating the meaning of the relationships between components are needed. For instance, quantitative studies can be conducted to test how these relationships exactly are (e.g., direction, intensity) and how they change over time with for instance longitudinal studies and corresponding data analyses. In qualitative studies, participants can, for example, be asked to explicitly name relationships between components or depict them as semantic networks which leads to a more in-depth understanding of the relationships between the components. Moreover, to capture processes there are more possibilities. In addition to correlations between variables, and longitudinal studies to capture processes over time, more fine-grained information and insights into processes are required where for instance data analytics can be of use (e.g., Van de Ven and Poole, 2005). Studies so far did not capture all components of reflection processes (e.g., Hetzner et al., 2011; Anderson, 2020). An example is the focus on the relationship between perceived usefulness of professional development (as an individual factor) and reflective thinking (Welp et al., 2018). Furthermore, qualitative research could be used to gain a more in-depth understanding of the meaning of the relationships between the components (Mulder, 2022). This also accounts for the relationships between emotions, motivation, and cognition within the intrapersonal processes. In addition to getting more insight into the complexity, dynamics of these processes might be investigated more thoroughly. Considering reflection as a process of change would provide opportunities for further, relevant, and helpful studies, also by using other forms of instruments and data, such as data analytics (Van de Ven and Poole, 2005).

### Practical implications

As previously mentioned, this conceptual model can be used by researchers to increase the understanding of reflection processes at work in different domains. Furthermore, it can be used by employees as tool to analyze their own reflection processes. Guiding questions could be added for this purpose (Korthagen and Vasalos, 2005). Firstly, because the model emphasizes that cognition, motivation, and emotion play a role during the processes which can increase the awareness of employees on the complexity of reflection processes and can help them to analyze their own reflection processes. Secondly, by using this model as a framework a person can analyze their own reflection processes in terms of discovering the relationships between triggers, context, individual factors with reflection processes and the outcomes. These so gained new insights can be used to develop informal and formal measures (e.g., training, coaching, asking colleagues for feedback) to improve next reflection processes and consequently the outcomes for instance in terms of competences.

The use of this model as an analysis tool can also help others, such as leaders and people responsible for human resource development in organizations to increase insight in their own reflection, as well as to get insight into reflection processes of the employees. These insights can be used as a basis for the improvement of the reflection processes of all employees which fosters their professional development. In addition to the improvement of reflection as part of informal learning at work, these insights can be used as input for the development of more formal measures to increase professional development, such as training (e.g., Welp et al., 2018) or mentoring (e.g., Son, 2016).

In conclusion, the conceptual model integrates relevant components of reflection, captures the complexity and dynamics of reflection processes and can be used as a framework for analysing reflection at work in all kinds of domains. The results of such analyses enable fostering reflection and improving reflection processes as part of professional development in concrete work situations.

## Ethics statement

Ethical review and approval was not required for the study on human participants in accordance with the local legislation and institutional requirements. The patients/participants provided their written informed consent to participate in this study.

## Author contributions

All authors listed have made a substantial, direct, and intellectual contribution to the work and approved it for publication.

## Conflict of interest

The authors declare that the research was conducted in the absence of any commercial or financial relationships that could be construed as a potential conflict of interest.

## Publisher’s note

All claims expressed in this article are solely those of the authors and do not necessarily represent those of their affiliated organizations, or those of the publisher, the editors and the reviewers. Any product that may be evaluated in this article, or claim that may be made by its manufacturer, is not guaranteed or endorsed by the publisher.
